# Cardiac Arrhythmia Classification by Multi-Layer Perceptron and Convolution Neural Networks

**DOI:** 10.3390/bioengineering5020035

**Published:** 2018-05-04

**Authors:** Shalin Savalia, Vahid Emamian

**Affiliations:** 1 Department of Electrical Engineering, St. Mary’s University, 1 Camino Santa Maria, San Antonio, TX 78228, USA; 2 School of Science, Engineering and Technology, St. Mary’s University, San Antonio, TX 78228, USA; vemamian@gmail.com

**Keywords:** electrocardiogram (ECG), arrhythmia, deep neural network, machine learning, deep learning, PhysioBank, kaggle, python, TensorFlow

## Abstract

The electrocardiogram (ECG) plays an imperative role in the medical field, as it records heart signal over time and is used to discover numerous cardiovascular diseases. If a documented ECG signal has a certain irregularity in its predefined features, this is called arrhythmia, the types of which include tachycardia, bradycardia, supraventricular arrhythmias, and ventricular, etc. This has encouraged us to do research that consists of distinguishing between several arrhythmias by using deep neural network algorithms such as multi-layer perceptron (MLP) and convolution neural network (CNN). The TensorFlow library that was established by Google for deep learning and machine learning is used in python to acquire the algorithms proposed here. The ECG databases accessible at PhysioBank.com and kaggle.com were used for training, testing, and validation of the MLP and CNN algorithms. The proposed algorithm consists of four hidden layers with weights, biases in MLP, and four-layer convolution neural networks which map ECG samples to the different classes of arrhythmia. The accuracy of the algorithm surpasses the performance of the current algorithms that have been developed by other cardiologists in both sensitivity and precision.

## 1. Introduction

**Figure 1:**
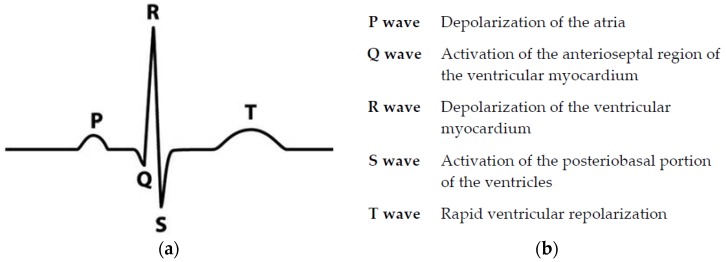
Ideal electrocardiogram (ECG) signal with key features indicated; (**a**) P wave, QRS complex, and T wave which play important roles in diagnosis abnormality of heart signal; (**b**) Features of an ECG signal; how and which part of heart is used to generates each feature [16].

Electrocardiography (ECG) is a procedure used to evaluate the electrical activity of the heart with reference to time by insertion of electrodes on the skin. The electrodes can recognize trivial electrical changes in skin. ECG detects physical cardiac activities which are shaped by the re-polarization and depolarization of the atria and ventricles of the heart. Heart signals consist of several features such as P waves, QRS complex, and T waves, and studying such features plays an imperative part in the diagnosis of various arrhythmias [1]. Figure 1 shows an ECG signal with a description of its key features. Studies of such features focus on detecting and classifying various types of arrhythmias, which can be described as an irregular heart rate or irregular features of the signal. Previously, the focus of our research was on “classification of cardiovascular diseases by using feature extraction and artificial neural networks” which intended to discriminate normal and abnormal ECGs by using artificial neural networks and subsequently extract the various features of the signal by using a state-logic machine algorithm which could detect certain cardiac diseases, such as tachycardia, bradycardia, and first and second-degree AV (Atrioventricular) block. There are other arrhythmias that are emphasized in this research, such as ventricular tachycardia, atrial flutter, atrial fibrillation, malignant ventricular, and ventricular bigeminy with the help of deep neural network algorithms.

Different approaches have been recently presented for automatic identification of ECG arrhythmia based on signal feature extraction, such as support vector machine (SVM) [2,3], discrete wavelet transform (DWT) [4,5], feed forward neural network (FFN) [6], learning vector quantization (LVQ) [7,8], back propagation neural network (BPNN) [9], and regression neural network (RNN) [10]. When a large number of datasets is available, deep learning models are a good to approach and often surpass human agreement rates [11]. CNN was used for automated detection of coronary artery disease and it was found that CNN remains robust despite shifting and scaling invariance which makes it advantageous [12]. In this research, the authors propose robust methods for cardiac disease diagnosis by using CNN and multilayer perceptron (MLP). CNN was also used to distinguish normal/abnormal heart sound recordings with accuracy of 82% which is reliable for large datasets [12]. The deep learning method for single-image super-resolution (SR) was also developed using a CNN method with superior performance than the state-of-the-art method [13]. In the 2017 PhysioBank competition, Fernando et al. proposed an algorithm with an accuracy of 83% on test data, which uses CNN to identify four different arrhythmias from short segments of ECG recordings [14]. In the same competition, Ghiasi et al. detected atrial fibrillation using a feature-based algorithm and deep CNN with 80% accuracy on training datasets [15].

**Figure 2:**
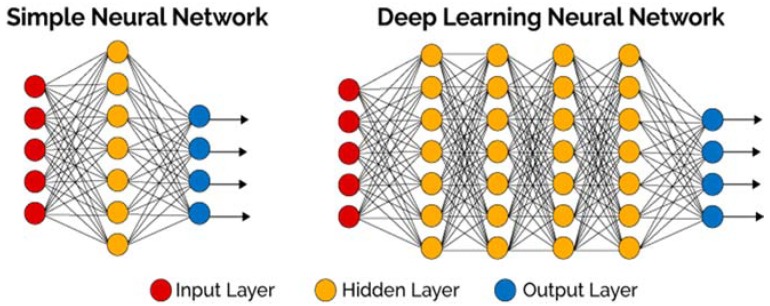
Comparison between simple neural network (NN) and deep NN; simple neural networks contain only one hidden layer as well as the input and output layers, while deep learning neural networks contain more than one hidden layer. In this case, there are four hidden layers between the input and output layers [17].

Traditional machine learning algorithms only use input and output layers, and at most a single hidden layer. Use of more than three layers (including input and output) is referred to as “deep” learning” [17]. Figure 2 distinguishes between simple NN and deep learning NN. The main benefit of DNN (Deep Neural Network) is that it can recognize more complex features because of the number of hidden layers it contains. This function of DNN makes it capable to handle large, high-dimensional data which contains a large number of features. Deep learning networks end in an output layer: a logistic, or softmax, classifier that assigns a likelihood to a particular outcome or label [17].

In the proposed algorithms, two PhysioBank datasets (normal sinus rhythm database (NSR-DB) and MIT/BIH arrhythmia database) were used to distinguish normal and abnormal ECG signals, for which the multilayer-perceptron technique was used. Another algorithm uses a four-layer of convolution neural network (CNN) to detect various arrhythmias in arbitrary length ECG dataset features. The dataset that was used in this study contains various cardiac diseases, such as arrhythmia, normal sinus, second degree AV block, first degree AV block, atrial flutter, atrial fibrillation, malignant ventricular, ventricular tachycardia, and ventricular bigeminy. It was downloaded from kaggle.com. The models were trained with help TensorFlow library developed by Google in 2015 specifically for machine learning and deep neural networks. Once both models had been trained on the downloaded ECG dataset, they were trained with another dataset with different characteristics from the training dataset.

## 2. Methodology

### 2.1. Problem Formulation

The algorithm for detection of ECG arrhythmias is a sequence-to-sequence task which takes an input (the ECG signal) *S* = [*s_1_*, …, *s_k_*] and gives labels as an output in the form of *r* = [*r_1_*, …, *r_n_*], where each *r_i_* can take any of m different labels. For the multilayer perceptron algorithm, m = 2, and for the CNN algorithm, m = 9. The individual output label corresponds to a segment of the input. Composed output labels cover the full sequence [18].

For a solitary example in the training set, we enhance the cross-entropy function;

(1) $$L\left(S,r\right)=\frac{1}{n}\overset{n}{\underset{i=1}{\sum }}\log p\;(R=r_{i}\;|\;S)$$

where *p* is the probability the network assigns to the *i*th output, taking on the value *r_i_*.

### 2.2. Convolutional Neural Network

Convolutional neural networks were first developed by Fukushima in 1980, and then in later years was improved [18]. It is a form of DNN which comprises one or more convolutional layers followed by one or more fully connected layers as in a standard multilayer neural network. The main advantages of CNNs are that they are easier to train and have fewer parameters than fully connected networks with the same number of hidden layers [18]. CNNs are self-learned and self-organized networks which eliminates requirements of supervision. Nowadays, an important application of CNN is in image classification, object recognition, and handwriting recognition. In addition, it plays an important role in the medical field for automated disease diagnosis.

Whereas some machine learning algorithms ask for pre-processing of datasets and separate feature extraction techniques, CNN does not have these requirements. This makes CNN advantageous and reduces liability during training and picking of the best feature extraction procedure for the automatic detection of arrhythmias [18,19]. 

### 2.3. Multilayer Perceptron

MLP is one of the main branches of feedforward artificial neural networks. MLP consists of a minimum of three layers of nodes. MLP utilizes the backpropagation technique for its training which is part of the supervised learning method [19]. This structure of deep learning is able to distinguish data which are not linearly separable.

Whenever data is linearly separable, all neurons can have a linear activation function, which will linearly map the input to the output. For non-linearly separable data, the algorithm will use a non-linear activation function, such as a sigmoidal or logistic function [20]. MLP is very popular in diverse fields, such as speech recognition, image recognition, and machine translation software.

### 2.4. Model Architecture

**Figure 3:**
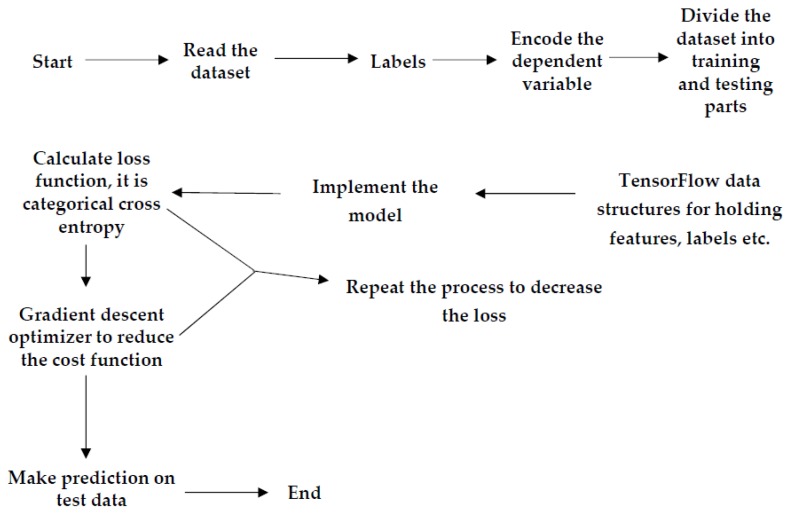
System process flowchart of Multilayer Perceptron (MLP) and Convolution NN. To define features and labels in the dataset, two TensorFlow variables were defined. One hot encoder was used to encode the dataset.

**Figure 4:**
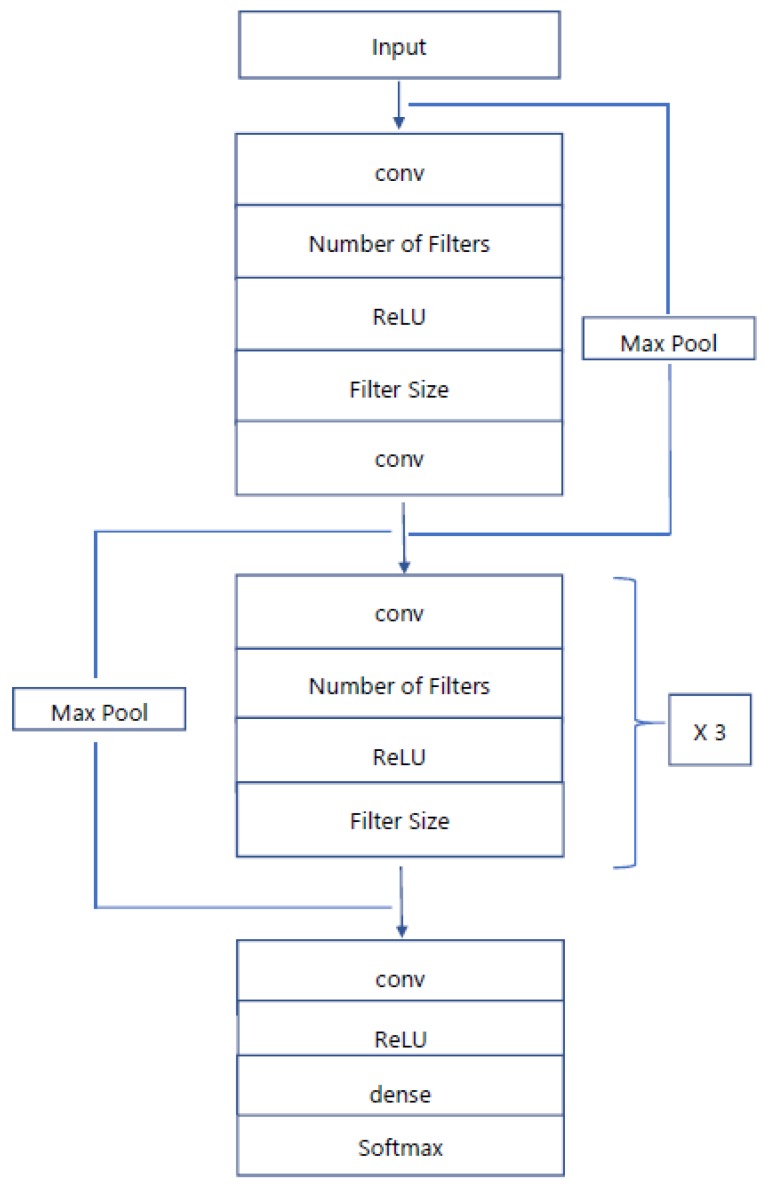
Proposed Algorithm of Convolution NN. Four convolutional layers were used. In addition, there is one output layer with a softmax layer to hold the output of the NN.

Algorithms use convolutional neural networks and multilayer-perceptron with a number of hidden layers used for sequence-to-sequence learning tasks. The convolutional neural network is one of the central branches of deep, feed-forward machine learning artificial neural networks that can handle large amounts of data and visual imagery. As with normal DNN, CNN has input, output, and a number of hidden layers. The hidden layers of CNNs mainly comprise convolutional layers, pooling layers, fully connected layers, normalization layers, and softmax layers. The proposed CNN algorithm has a convolutional layer with softmax function which provides the output of the trained network. The algorithm uses the rectifier linear unit (ReLU) activation tool in all convolution layers. The max pooling layer works independently for each row and column of the input and spatially resizes it [21]. The max pooling layer with stride size of 2 × 2 was used in the algorithm because it gave better accuracy than a 3 × 3 pooling layer. Use of a 3 × 3 stride layer leads to high information loss. The pooling layer in the CNN reduces the overfitting problem by making the input size half of the actual input. A flowchart of both algorithms is explained briefly in Figure 3. Both the models take features of an ECG signal as the input of the network and predict the output as labels of the signal. Initially, ECG datasets will be pre-processed. To do that, the first network reads the datasets, and then defines their features and labels. In the MLP algorithm, the labels will be arrhythmia and normal sinus, while in the CNN algorithm, the labels are arrhythmia, normal sinus, second degree AV block, first degree AV block, atrial flutter, atrial fibrillation, malignant ventricular, ventricular tachycardia, and ventricular bigeminy [22]. Figure 4 explains the proposed architecture of the CNN in the algorithm where the first and last convolutional layers are different from the middle three convolutional layers.

The next step is to encode the dependent variable—the dataset labels—for the deep network. As the dataset is categorical, containing different arrhythmia names as labels, it is mandatory to encode the dataset because the labels are not numerical and cannot be read directly by the algorithm [23]. There are two statistical methods for encoding data; one is integer encoding and other is one-hot encoding. Integer encoding will assign an integer value to each unique category value. For example; “red” is 1, “green” is 2, and “blue” is 3 [23]. For categorical variables where no such ordinal correlation exists, integer encoding is not sufficient. In one-hot encoding, the integer encoded variable is removed and a new binary variable is added for each unique integer value. In the “color” variable example, there are 3 classes and consequently 3 binary variables are needed. A “1” value is placed in the binary variable for the color and “0” values are used for the other colors. In the proposed machine learning algorithms, one-hot encoding was used to avoid conflicts of integer encoding. This was followed by dividing the dataset into three parts; for training, testing, and validation [23].

In the following step, the TensorFlow data structures was defined for holding features, labels etc., which includes defining weights, biases, hidden layers, activation tools, filters, filter size, placeholders for inputs, and desired output. There is also another tensor defined to store trained model output. This was followed by implementation and training of the model with the training dataset. Once the network is trained, it will calculate how far the trained model’s output is from the actual output. Then, the cross-entropy function will try to reduce this error to a minimum point. Once it reaches the minimum value, the trained model will give testing accuracy by performing training with a test dataset [24].

### 2.5. ECG Data

**Figure 5:**
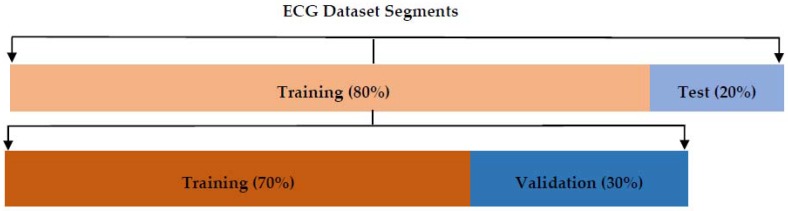
The distribution of ECG segments used for training and testing. Eighty percent of the data was used for training and 20% was used for testing. Thirty percent of the training dataset was used for validation of the network.

ECG data that was downloaded from PhysioBank.com and kaggle.com was used for the MLP and CNN algorithms, respectively, for training and testing. The MLP dataset had dimensions of (208, 61), where the 208 rows are the total ECG signals and the 61 columns are the total number of features and labels. The first 60 columns contain features, whereas the last column contains the label (diseases) of each individual signal. On the other hand, the CNN dataset had dimensions of (26,543, 60), following the same pattern as the MLP dataset, but this dataset contained 9 different labels. Both the algorithms used 80% of the total data for training and 20% for testing. Furthermore, the training dataset was divided into 70% for actual training and 30% for validation. Each ECG signal in the dataset was 10 s long and contained one rhythm class. An illustration of the distribution of ECG signals used for training, testing, and validation procedures can be seen in Figure 5.

### 2.6. Training of Data

In the training part, a batch size of 50 was used with the standard back propagation algorithm for stochastic learning. The formula that was used to update the weights is as follows [25];

(2) $$w_{l}=\left(1-\frac{n_{\lambda }}{ts}\right)\;w_{l-1}-\frac{n}{x}\frac{\partial c}{\partial w}$$

where w = weights; *l* = layer number; n = learning rate; λ = regulation parameter; ts = total number of training samples; x = batch size; *c* = cost function.

In addition, the biases are updated through,

(3) $$b_{l}=b_{l-1}-\frac{n}{x}\frac{\partial c}{\partial w}$$

In the proposed algorithms for the deep neural networks, the learning rate was defined as 0.002 for MLP and 0.003 for CNN.

### 2.7. Testing of Data

After completion of each training epoch, the algorithms will perform testing on the CNN and MLP models to give test accuracy. Keep in mind that the MLP and CNN algorithms have 1000 and 500 epochs, respectively. Thirty percent of the total training data (80% of the original dataset) was used as the validation part and was used after completion of every epoch to improve accuracy. As shown earlier in the distribution of the ECG signals for training, testing, and validation, 20% of the total data was used for testing [26].

## 3. Results

**Figure 6:**
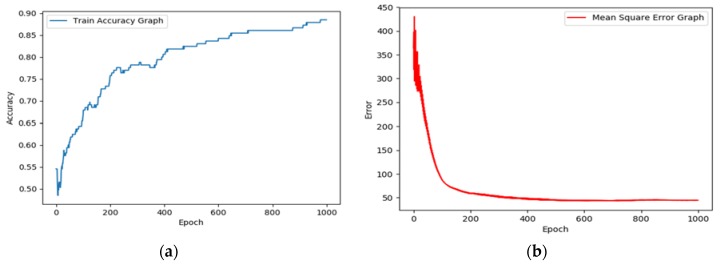
Accuracy and mean square error (MSE) graph of the MLP algorithm; (**a**) the accuracy of MLP increases as the number of epochs increases; (**b**) MSE reduces with every epoch and reaches the minimum point after 1000 epochs.

Convolution neural networks have the remarkable ability to extract all the dissimilar features which are relatively invariant to local spectral and temporal variations, and this has resulted in many breakthroughs in higher accuracy results. Basically, the CNN algorithm contains three parts: (1) data preprocessing of input; ECG signals are processed, after which the computer can understand different diseases, (2) stacking of convolution layers and max pooling layers to extract the features, (3) layering of a fully connected layer and activation of the softmax function which will predict the disease [16]. Table 1 gives the parameters of the CNN layers and their filter size and output neuron size. The MLP algorithm was used to distinguish between normal sinus rhythm and abnormal rhythm. For this, four hidden layers were used, and each layer consisted of 60 neurons. The ReLU function was used to activate the first and last hidden layers, whereas the two middle hidden layers use a sigmoidal activation function. This was followed by the linear activation function in the output layer. In addition, a gradient descent optimizer was used to reduce the error between the trained network output and the actual output. It is advantageous to implement a gradient descent optimizer when the parameters cannot be calculated analytically or by linear algebra. Figure 6 shows the accuracy graph and MSE (Mean Square Error) graph of the MLP algorithm.

**Figure 7:**
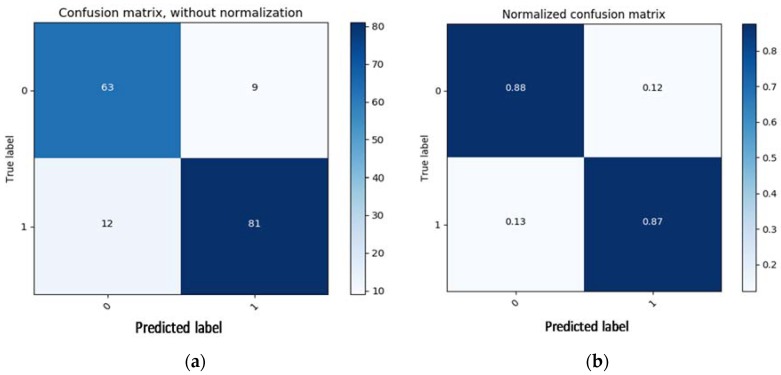
Confusion matrix (CM) with and without normalization of the MLP algorithm; (**a**) 63 arrhythmias and 81 normal signals are correctly classified, while 9 arrhythmias and 12 normal ECG signals are misclassified; (**b**) CM with normalization gives an accuracy in percentage; in this case, accuracy was 88% for arrythmia and 87% for normal sinus ECG.

Once the network was trained with 1000 epochs, it gave an accuracy of 88.7% for the PhysioBank.net dataset. Figure 7 shows the visual confusion matrix for the training part of the dataset. The confusion graph is a plot of true label versus predicted label, where 0 stands for abnormal ECG signal and 1 represents normal sinus rhythm. The network outputs are accurate, as shown by the high number of correct responses in the blue squares and the low number of incorrect responses in the white squares. The dataset consists of a total of 208 ECG recordings, 97 of which are abnormal (arrhythmia) and 111 represent a normal sinus rhythm. As mentioned previously, 80% of the data was used for training, constituting 165 ECG signals, 72 of which represent arrhythmia and 93 represent normal sinus rhythm. Of this training data, 63 arrythmia and 81 normal sinus signals were correctly classified by the algorithm, an improvement in the accuracy of the MLP model.

**Figure 8:**
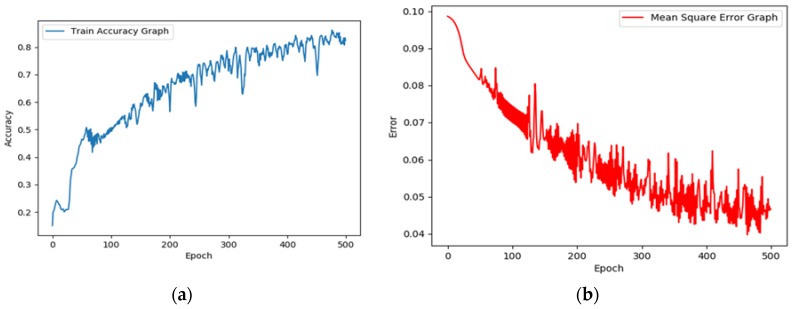
Accuracy and MSE of the CNN algorithm; (**a**) accuracy of the CNN rises continuously with every epoch; (**b**) MSE of CNN reduces with each epoch.

In next section, the deep neural network created as a convolution neural network to identify various cardiovascular diseases. The ReLU non-linear activation tool was used to activate the CNN along with the gradient descent optimizer which will minimize the error of network. This tool becomes very beneficial when the parameters cannot be calculated analytically (i.e., using linear algebra) [27]. As discussed earlier in the architecture of the CNN algorithm, each convolution layer has 32 filters and each filter has dimensions of 5 × 5. Figure 8 graphically shows the accuracy and MSE. The accuracy increases constantly with every epoch and after 500 epochs, reaches a value of 83.5%. Furthermore, the MSE reduces constantly with each epoch and at the end it reaches a minimum point.

**Figure 9:**
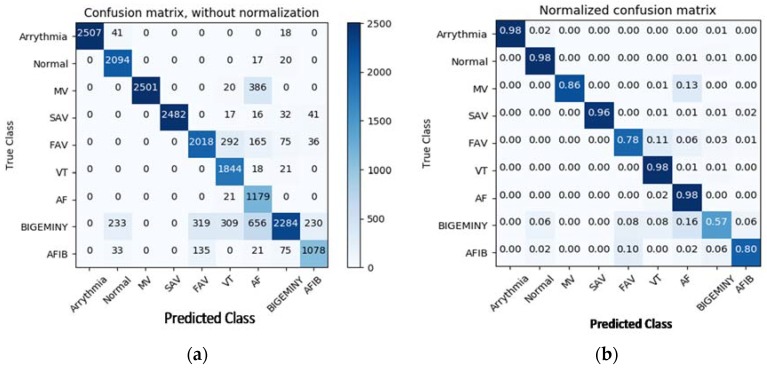
CM with and without normalization of the CNN algorithm; (**a**) most of the arrhythmia was correctly classified by CNN except bigeminy and FAV; those diseases might have the same characteristics as others; (**b**) normalized CM gives the accuracy of the CNN in percentage form.

After defining two variables as the features and labels of the datasets, the algorithm will reshape the dimensions of the features by 1 × 4 because the convolution layer only accepts 4-dimension arrays [28,29,30,31]. Upon completion, the first, second, and third convolution layers are defined, where the output of first layer will feed into the max pooling layer which will reduce the dimension of the array to make the network faster and avoid overfitting. This same organization follows for the second and third convolution layers too. The result of the third pooling layer will feed into the fully connected layer, followed by the softmax layer where the network will predict the diseases [32]. The classification results of the system are exhibited by using a confusion matrix. In a confusion matrix, each cell comprises the raw number of exemplars classified for the matching combination of desired and actual network outputs. Figure 9 gives a visual representation of the confusion matrix for the CNN algorithm. Many arrhythmias were confused with first-degree AV Block (FAV) and ventricular bigeminy, but other than that, the network gives respectable prediction accuracy for the other diseases. We expect that part of this is due to the sometimes-ambiguous location of the exact onset and offset of the arrhythmia in the ECG recording [33,34,35].

## 4. Conclusions

In this research, we developed a diagnosis system for identifying various cardiovascular diseases using deep learning methods. Generally, ECG arrhythmia can be easily identified from its shape. Due to the prevalence of serious arrhythmias, there is a need to develop a well-organized and robust CAD (computer aided design) system to accurately and automatically detect several types of arrhythmias. The proposed algorithms were tested on ECG signals obtained from Physio.net and keggar.com. These constitute real ECG signals collected from patients for medical research. The algorithms succeeded in detecting all disease states in each signal with significant accuracy by using MLP and CNN models (Table A1). The MLP algorithm uses four hidden layers and the CNN uses four convolution layers. In CNN algorithm, two diseases, first-degree AV block (FAV) and ventricular bigeminy, have significant misprediction. These diseases might have some similarity in their features with other diseases, leading to confusion of the network. The stated results show that the proposed algorithms can make efficient diagnoses of various cardiovascular diseases with 88.7% accuracy for MLP and 83.5% for CNN. Although the performance of the anticipated methods is decent, the problem of arrhythmia diagnosis is far from being solved. There are many complications worth investigating. According to our research, bigeminy features are easily mistaken for normal, FAV, VT, AF, and AFIB signals, which would lead to false positives. Deep learning is the most promising direction for cardiac abnormality detection and more investigations are still needed in that direction.

## Figures and Tables

**Table 1:** Details of the proposed CNN algorithm with description of filter size and number of neurons used for each convolution and max pooling layer.

Layers	Type	Size of Neurons (Output Layer)	Filter Size of Each Layer
0–1	Convolution	(None, 1, 60, 1)	32
1–2	Max Pooling	(None, 1, 30, 1)	2
2–3	Convolution	(None, 1, 30, 1)	32
3–4	Max Pooling	(None, 1, 15, 1)	2
4–5	Convolution	(None, 1, 15, 1)	32
5–6	Max Pooling	(None, 1, 8, 1)	2
6–7	Convolution	(None, 1, 8, 1)	32
5–6	Fully connected layer	2048	-

## Appendix A

**Table A1:** A list of all arrhythmia types which the model classifies. For each arrhythmia, we give the label name, a more descriptive name, and an example chosen from the training set. We also give some description of each arrhythmia type [23].

Class	Description	Example
Normal	Normal Sinus Rhythm means normal heart rate, in respect to both heart rate and rhythm. Heart Rate—60 to 100 BPM	
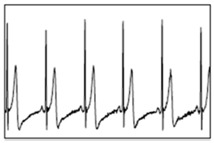
VT	Ventricular Tachycardia is heart rhythm illness instigated by abnormal signals in the lower chambers of the heart. Heart Rate—More than 100 BPM	
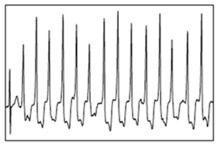
AFIB	Atrial Fibrillation is an irregular and fast heart rate than can increase chance of stroke, heart failure. Heart Rate—100 to 175 BPM	
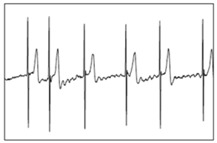
AF	Atrial Flutter is the same as AFIB. But, whereas AFIB causes increased heart rate without a regular pattern, AFL causes increased heart rate in a regular pattern. Heart Rate—100 to 175 BPM	
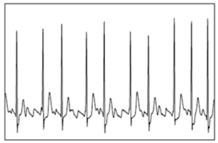
SAV	Second Degree AV, is a disease of the cardiac conduction system in which the conduction of atrial impulse over the AV node and/or his bundle is delayed or blocked.	
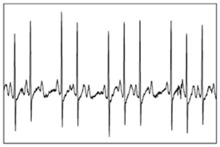
Bigeminy	Ventricular Bigeminy is a heart rhythm problem in which there is a continuous alternation of long and short heart beats.	
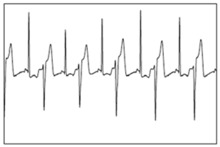
FAV	In First Degree AV Block conduction is slowed, there are no missed beats. In first-degree AV block, every atrial impulse is transmitted to the ventricles, resulting in a regular ventricular rate.	
